# Accuracy of clinical diagnosis of behavioral variant frontotemporal dementia: A systematic review and meta‐analysis

**DOI:** 10.1002/dad2.70266

**Published:** 2026-02-28

**Authors:** Daniele Urso, Stefano Giannoni‐Luza, Silvio Ionta, Stefania Mondello, Giancarlo Logroscino

**Affiliations:** ^1^ Center for Neurodegenerative Diseases and the Aging Brain, Department of Clinical Research in Neurology University of Bari “Aldo Moro,” “Pia Fondazione Cardinale G. Panico” Tricase Italy; ^2^ SensoriMotorLab, Department of Ophthalmology/University of Lausanne, Jules Gonin Eye Hospital/Fondation Asile des Aveugles Lausanne Switzerland; ^3^ Department of Biomedical and Dental Sciences and Morphofunctional Imaging University of Messina Messina Italy; ^4^ Department of Translational Biomedicine and Neuroscience (DiBraiN) University of Bari “Aldo Moro Bari Italy

**Keywords:** accuracy, frontotemporal dementia, meta‐analysis, sensitivity, specificity, systematic review

## Abstract

**INTRODUCTION:**

Accurate diagnosis of behavioral variant frontotemporal dementia (bvFTD) remains difficult due to overlapping symptoms with other conditions. We aimed to evaluate the diagnostic accuracy of bvFTD clinical criteria and whether it has improved over time.

**METHODS:**

We systematically reviewed studies assessing the Lund‐Manchester (1994), Neary (1998), and Rascovsky (2011) criteria, using pathology or follow‐up diagnosis as reference standards. A Bayesian bivariate model was used to estimate pooled sensitivity and specificity.

**RESULTS:**

Nine studies (1130 participants) were included. Sensitivity increased from 0.70 (95% confidence interval [CI], 0.41–0.89; Lund‐Manchester) to 0.80 (95% CI, 0.48–0.95; Neary) and 0.90 (95% CI, 0.81–0.95; Rascovsky, probable diagnosis), at the cost of a decreased specificity (0.94, 0.95, and 0.85, respectively).

**DISCUSSION:**

The diagnostic sensitivity of bvFTD criteria has improved over time, though specificity has declined. Ongoing refinement and biomarker development are needed to enhance diagnostic accuracy, especially in early disease stages.

## Highlights

- Sensitivity of behavioral variant frontotemporal dementia (bvFTD) clinical criteria improved from 70% to 90% over time.
- Psychiatric and Alzheimer's disease misdiagnoses were most frequent sources of error.
- Pathology‐based diagnoses yielded higher sensitivity than clinical follow‐up.
- Updated criteria enhance bvFTD detection, but specificity needs refinement.

## 1 INTRODUCTION

Behavioral variant frontotemporal dementia (bvFTD) is a complex neurodegenerative disorder associated with progressive changes in behavior, personality, and social functioning.^1^ This condition is part of the broader category of frontotemporal dementia (FTD), encompassing a spectrum of disorders with unique clinical and neuropathological profiles involving proteinopathies associated with frontotemporal network dysfunction.^2^, ^3^, ^4^, ^5^ Individuals with bvFTD experience an early‐onset deterioration in cognitive and emotional faculties, leading to significant changes in personality and behavior, including behavioral disinhibition, apathy or inertia, loss of sympathy or empathy, perseverative and stereotyped or compulsive and ritualistic behavior, and hyperorality and dietary changes.^6^ Although once considered rare and predominantly affecting those with young‐onset dementia, recent findings from a multinational European study suggest that bvFTD is more common than previously described and should be considered even in the elderly, beyond the age of 70 years.^7^

Accurate diagnosis of bvFTD is crucial for patients, their families, and health‐care providers.^8^ Unlike Alzheimer's disease (AD), for which robust imaging and laboratory biomarkers exist, and Lewy body dementia, for which emerging biomarkers reflect the disease's pathophysiology, such valuable tools are not yet available for FTD.^9^ Hence, clinical criteria remain essential for identifying individuals with bvFTD and aiding in medical decision making.^10^ An early accurate diagnosis is pivotal for prognosis, timely initiation of treatment and its planning, and patient management.^11^, ^12^, ^13^ This also offers invaluable information to family members and caregivers, who often experience significant stress and burden.^8^

However, diagnosing bvFTD presents challenges due to the subtlety of behavioral and personality changes, which can mimic other neurodegenerative disorders or psychiatric conditions.^14^, ^15^ The diagnostic complexity is further compounded by the diverse pathological substrates underlying bvFTD, hindering straightforward diagnosis.^16^ Over the years, diagnostic criteria for bvFTD have been revised to overcome these challenges and improve accuracy.^6^, ^17^, ^18^ Yet, uncertainty remains regarding the discriminative accuracy of the different set of clinical criteria, which is an essential step to define their clinical utility. We therefore conducted a systematic review and meta‐analysis to determine the diagnostic accuracy of the frequently used diagnostic criteria for bvFTD (Lund‐Manchester 1994 criteria,^19^ Neary et al. 1998 criteria,^18^ and the international consensus criteria for behavioral variant FTD [FTDC] proposed by Rascovsky et al. in 2011^6^) and compare their different performance.

## 2 METHODS

This systematic review and meta‐analysis was conducted in accordance with our published protocol^20^ (PROSPERO‐CDR42023389063) and followed the Cochrane handbook^21^ and the Preferred Reporting Items for Systematic Reviews and Meta‐Analyses (PRISMA) reporting guidelines^22^ (Table S1 in supporting information).

### 2.1 Study types and eligibility criteria

We included peer‐reviewed articles of observational studies reporting information related to the clinical diagnosis of bvFTD by a well‐defined set of criteria as an index test and supported by a well‐defined reference standard. The index test consisted of three generations of bvFTD clinical diagnostic criteria: (1) Lund‐Manchester 1994 criteria,^19^ (2) Neary et al. 1998 criteria,^18^ and (3) the FTDC proposed by Rascovsky et al. in 2011.^6^ As reference standards, we considered pathology and genetic diagnosis as the primary gold standards, but also included refined diagnosis by experts after a follow‐up period. Eligible studies were required to provide complete or partial data on diagnostic performance measures—sensitivity (proportion of patients with bvFTD correctly diagnosed using clinical criteria), specificity, positive predictive value (PPV), negative predictive value (NPV) = allowing the reconstruction of 2 × 2 tables (true positive [TP], false positive [FP], true negative [TN], and false negative [FN]). We excluded abstracts, book chapters, and reviews, and studies that did not specify the diagnostic clinical criteria were also excluded. Studies were also excluded if they failed to meet the inclusion criteria or if essential information was missing and could not be obtained from the authors. No restriction was placed on population, setting, publication year, or language.

**RESEARCH IN CONTEXT**

1. **Systematic review**: We performed a systematic review and meta‐analysis following a preregistered protocol. Searches were conducted in MEDLINE, Embase, Web of Science, and LILACS to identify studies evaluating the diagnostic accuracy of behavioral variant frontotemporal dementia (bvFTD) clinical criteria (Lund‐Manchester, Neary, and Rascovsky). Only studies using pathology or expert clinical diagnosis after follow‐up as reference standards were included.
2. **Interpretation**: Our findings demonstrate that the sensitivity of bvFTD clinical diagnostic criteria has improved over time, from 70% with Lund‐Manchester to 90% with probable Rascovsky criteria. This improvement, however, is accompanied by decreased specificity. The narrowing of confidence intervals for likelihood ratios suggests increased precision and clinical utility of the updated criteria.
3. **Future directions**: Future research should prioritize validation of biomarkers that improve specificity, investigate bvFTD phenocopies, and assess diagnostic performance across diverse populations and non‐tertiary settings. Studies using harmonized criteria and pathology‐confirmed cohorts remain crucial.

### 2.2 Search strategy and study selection

We performed an electronic literature search on May 1, 2024, in four databases: MEDLINE‐PubMed, Web of Science, Embase, and LILACS (full search strategy is shown in Table S2 in supporting information). It consisted of a combination of terms mainly as title/abstract and medical subject headings including terms related to bvFTD (e.g., frontotemporal lobar degeneration [FTLD]) and diagnostic accuracy (e.g., sensitivity) tailored to each database. The screening and selection process were performed using the systematic review manager COVIDENCE (Veritas Health Innovation, Melbourne, Australia). After removing the duplicates, two authors (D.U., and S.G.‐L.) independently undertook screening of titles and abstracts, followed by full‐text review of retained articles. Discrepancies were resolved by consensus with a third researcher (S.M.). Reasons for exclusion at the full‐text phase are shown in Table S3 in supporting information. In addition, we carefully reviewed the list of references for additional articles missed in our search strategy.

### 2.3 Data extraction and preparation

Two reviewers (D.U. and S.G.‐L.) independently collected study data using a standardized spreadsheet. We extracted (1) general characteristics from the included studies (i.e., title and authors of the study, year of publication, journal, country, study design and funding, sample size, time of recruitment, population setting), (2) patient demographics (i.e., age, sex, comorbidities), (3) clinical characteristics (i.e., disease duration, imaging findings, genetic variants), (4) reference standard, (5) diagnostic criteria (index test), and (6) relevant outcome data (i.e., sensitivity, specificity, accuracy, PPV, NPV, TP, TN, FP, and FN values). For the FN and FP, we also extracted the specific misdiagnoses. In case any relevant information was missing or needed to be clarified we contacted the authors by e‐mail.

### 2.4 Risk of bias assessment

We tailored the Quality Assessment of Diagnostic Accuracy Studies (QUADAS‐2) tool^23^ (Table S4 in supporting information) and used it to classify the risk of bias and applicability concerns from the included studies in high, unclear, or low risk. The QUADAS‐2 evaluates the risk of bias related to four domains: patient selection, index test, reference standard, and flow and timing, while only the first three domains are assessed for applicability concerns. Two authors (D.U., S.G.‐L.) independently evaluated each article and disagreements were resolved by a third researcher (S.M.).

### 2.5 Statistical analysis and data synthesis

Two‐by‐two tables were constructed to calculate the sensitivity and specificity for each study with their 95% confidence intervals (CIs). We calculated summary sensitivity and specificity points using Bayesian random effects bivariate models to account for the unexplained heterogeneity between studies pairing the sensitivity and specificity through the meta‐analysis.^24^ In addition, positive and negative likelihood ratios (+LR and −LR, respectively) were calculated. Because the Rascovsky criteria is divided in two (i.e., possible and probable bvFTD), we calculated two models considering each of them separately. We meta‐analyzed the data when there were at least two studies. Individual and summary results were presented graphically in forest plots and receiver operating characteristic (ROC) plots. This last one displayed both the 95% confidence and predictive region for the summary point. We assessed heterogeneity by visually inspecting forest plots, and by ROC plots under the following intended subgroup analysis: reference standard type (pathologic and follow up), participant characteristics (age, disease duration), setting (clinic based vs. community based), and study design. We excluded studies classified as high in the risk of bias and/or applicability concerns sections from the QUADAS‐2 tool. In addition, we used the leave‐one‐out method to evaluate the influence of individual studies in the pooled sensitivity and specificity. According to the number of included studies, we intended to explore the presence of publication bias using a Deek funnel plot. Forest plots were created in R version 4.3.2, while the diagnostic accuracy analysis and ROC plots were performed in the web application MetaBayesDTA version v.1.5.2.^25^, ^26^, ^27^

## 3 RESULTS

**FIGURE 1:**
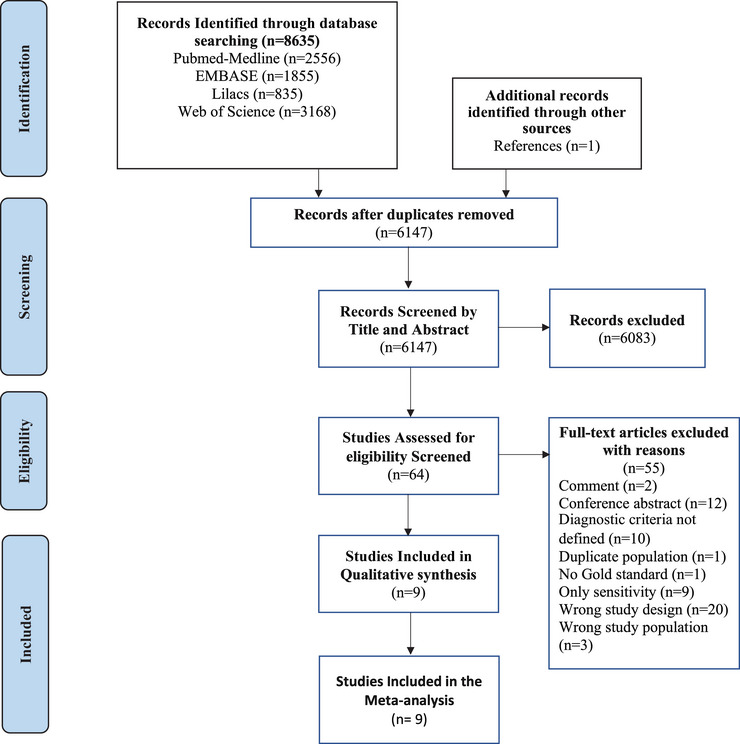
PRISMA flow diagram of the article search and selection process. Flow diagram illustrating the selection process of studies included in the systematic review and meta‐analysis. The diagram details the number of records identified through database searching, screened, excluded, and the final studies included in the review. Reasons for exclusion at each stage are specified, following the PRISMA guidelines. PRISMA, Preferred Reporting Items for Systematic Reviews and Meta‐Analyses.

Our search strategy retrieved 8635 articles. After removing duplicates, we screened 6147 titles and abstracts. We reviewed the full text of the remaining 64 studies, of which 55 were excluded. Reasons for exclusion at this stage can be found in Table S3. Finally, nine studies^28^, ^29^, ^30^, ^31^, ^32^, ^33^, ^34^, ^35^, ^36^ fulfilled our eligibility criteria and were included in our systematic review. A flow diagram of the search process is presented in Figure 1.

Among the included studies, seven had a diagnostic test accuracy design,^28^, ^30^, ^31^, ^32^, ^34^, ^35^, ^36^ one an agreement study,^29^ and one a cohort.^33^ Regarding the index test, two used the Lund‐Manchester,^28^, ^29^ three Neary,^30^, ^32^, ^34^ and four Rascovsky bvFTD diagnostic criteria.^31^, ^33^, ^35^, ^36^ As reference standards, five used pathology^28^, ^29^, ^34^, ^35^, ^36^ and four follow‐up.^30^, ^31^, ^32^, ^33^ All studies were conducted in a hospital setting and involved a total of 1130 patients with varied diagnoses, of which 383 (or 374 if the Harris et al. 2013 probable subset is used) corresponded to bvFTD confirmed by either pathology or refined diagnosis after follow‐up. Eight studies reported the proportion of patients by sex,^28^, ^30^, ^31^, ^32^, ^33^, ^34^, ^35^, ^36^ four reported the mean age of bvFTD onset,^28^, ^30^, ^31^, ^32^ five the disease duration,^28^, ^32^, ^33^, ^34^, ^35^ and three imaging^30^, ^31^, ^32^ and genetics^31^, ^33^, ^36^ results. Among them, ≈ 37% of patients were females, the mean age of onset was 57.67 ± 8.94 years, and the mean disease duration 3.6 years. None of the studies provided information related to race and ethnicity. A summary of the included studies’ characteristics can be found in Table 1. All except one study^32^ reported the misdiagnoses due to either FP, FN classification, or both. Among them, AD and psychiatric disorders were the most common misclassification causes (Table S5 in supporting information).

**TABLE 1:** Summary of included studies for qualitative assessment.

Study ID	Country	Study design	Start and end date	Population setting	Inclusion and exclusion criteria	Sample size	Age and sex no. (%)	Disease duration (years)	Pathologic confirmation(Autopsy/genetic)	Imaging findings	Clinical criteria
Lopez 1999^28^	USA	DTA	‐	Hospital	I: ‐Pathology consistent with dementia. E: ‐Not defined	40	67.78 ± 7.33 F: 19 (47.5)	3.2	Autopsy (*n* = 8) Genetic: NA	‐	Lund‐Manchester
Rosen 2002^29^	USA	Agreement	2009–2016	Hospital	I: ‐Neuropathological diagnosis of AD or FTLD. E: Not defined	60	—	—	Autopsy (*n* = 30) Genetic: NA	—	Lund‐Manchester
Mendez 2007^30^	USA	DTA	—	Hospital	I: ‐Referred patients for consideration of bvFTD. E: ‐Language‐predominant variants and (primary progressive aphasia or semantic dementia) and frontotemporal lobar degeneration.	134	63.41 ± 7.70 F:69 (51.5)	—	No pathology Re‐evaluation after follow‐up was the reference for comparison (*n* = 63) Genetic: NA	‐Frontotemporal atrophy in MRI (*n* = 61 of total possible FTD and 40 of the 63 FTD patients). ‐Predominant frontal, anterior temporal, or frontotemporal hypoperfusion or hypometabolism (*n* = 75 of total possible FTD and 57 of the 63 FTD patients).	Neary
Pijnenburg 2008^32^	The Netherlands	DTA	2002–2005	Hospital	I: ‐Subjects visiting the VU University Medical Center memory clinic. E: ‐Moderate to advanced dementia. ‐Obvious explanation for cognitive decline at first presentation other than neurodegenerative causes. ‐Clinical follow‐up < 1 year	193	60 (47–87) F:88 (36.26)	5	Autopsy: Only performed in one patient: FTLD‐UPS. Re‐evaluation after follow‐up was the reference for comparison (*n* = 19) Genetic: NA	Abnormal structural imaging consistent FTD (*n* = 10) Frontal/temporal deficit on functional imaging (F‐fluorodeoxyglucose PET or HMPAO SPECT) (*n* = 6)	Neary
Snowden 2011^34^	UK	DTA	1983–2008	Hospital	I: ‐Diagnosis of dementia in life. ‐Brain donated *post mortem* to Manchester Brain bank. ‐Full neuropathological examination. E: ‐Clinical diagnosis of Huntington's disease.	228	— 92 (40.35)	3	Autopsy (*n* = 92) Genetic: NA	—	Neary
Harris 2013^35^	UK	DTA	1979–2011	Hospital	I: ‐Consecutive patients investigated in specialist unit for early‐onset dementia plus *post mortem* neuropathological examination E: ‐Predominant primary progressive aphasia or extrapyramidal disorders.	156 total 146 (rated vs. possible bvFTD criteria) and 138 (rated vs. probable bvFTD criteria)128 (shared sample) *Overlap between groups (*n* = 128)	— F: 61 (41.8)	3.4	Autopsy/histopathology: Group rated versus possible bvFTD (*n* = 64): ‐FTLD tau(*n* = 25) ‐FTLD TDP‐43 (*n* = 35) ‐FTLD‐FUS (*n* = 2) ‐FTLD no inclusions (*n* = 2) Group rated versus probable bvFTD (*n* = 55): ‐FTLD tau (*n* = 24) ‐FTLD TDP‐43 (*n* = 28) ‐FTLD‐FUS (*n* = 2) ‐FTLD no inclusions (*n* = 1) Genetic: NA	—	Rascovsky
Balasa 2015^36^	Spain	DTA	2000–2013	Hospital	I: ‐Cases from NTB with *ante mortem* diagnosis of bvFTD. E: ‐Lack of adequate clinical information.	Total: 66	— F: 17 (25.8)	‐	Autopsy/histopathology: FTLD (*n* = 47): ‐FTLD‐tau (*n* = 18) ‐FTLD‐TDP (*n* = 26) ‐FTLD‐FUS (*n* = 2) ‐FTLD‐UPS (*n* = 1) Genetic: *MAPT* (*n* = 4) *PGRN* (*n* = 2) C9orf72 (*n* = 9)	—	Rascovsky
Vijverberg 2016^31^	The Netherlands	DTA	2011–2013	Hospital	I: ‐Behavioral symptoms dominated the presentation. ‐Score Frontal Behavioral Inventory ≥ 11 or Stereotypy Rating Inventory score was ≥ 10. E: ‐Benign bvFTD phenocopy. ‐No *post mortem* examination.	116	61.98 ± 6.85 F: 29 (25)	—	Histopathology: Tauopathy (*n* = 1) Genetic: ‐C9orf72 (*n* = 2) ‐GRN mutation (*n* = 1) Probable bvFTD at follow‐up (*n* = 27) Possible bvFTD at follow‐up (*n* = 27)	‐Frontal and/or temporal atrophy (*n* = 23 FTDC probable bvFTD) ‐Frontal and /or temporal hypometabolism frontotemporal on FDG‐PET (*n* = 20 FTDC probable bvFTD).	Rascovsky
De Boer 2023^33^	The Netherlands	Cohort	2011–2015	Hospital	I: ‐Participants in the LOF study (behavioral changes with onset between 45–75 years old)	137	— F: 37 (27.8)	3.5	Histopathology: Tauopathy (*n* = 1) Genetic: C9or72 expansion (*n* = 2) GRN mutation (*n* = 1) Probable bvFTD at follow‐up (*n* = 33)	—	Rascovsky

Abbreviations: AD, Alzheimer's disease; bvFTD, behavioral variant frontotemporal dementia; FTD‐UPS, frontotemporal dementia ubiquitin positive; DTA, diagnostic test accuracy; I, inclusion criteria; E, exclusion criteria; FTD, frontotemporal dementia; FTDC, frontotemporal dementia criteria; FTD‐FUS, frontotemporal dementia fused‐in‐sarcoma; FTD‐TDP, frontotemporal dementia transactive response DNA binding protein 43 kDa; FTD‐UPS, frontotemporal dementia ubiquitin–proteasome system; FTLD, frontotemporal lobar degeneration; HMPAO SPECT, exametazime s**ingle‐photon emission computed tomography**; LOF, late onset frontal lobe study; MRI, magnetic resonance imaging; NA, not available; NTB, neurological tissue bank; PET, positron emission tomography; UK, United Kingdom; USA, United States of America.

### 3.1 Risk of bias assessment

After the QUADAS‐2 assessment (Figure S1 in supporting information), two studies presented a high risk of bias^29^, ^32^ and one an unclear bias due to patient selection.^28^ Six studies had an unclear risk of bias for reference standard.^29^, ^30^, ^31^, ^32^, ^33^, ^34^ Regarding time and flow, only one study^32^ had unclear risk of bias due to uncertainty regarding the follow‐up periods undergone by the patients. The remaining articles presented a low risk of bias in the other categories. Regarding applicability concerns, only one study^29^ presented high concerns regarding patient selection. For the index test, there were low concerns, whereas for reference standard there were high applicability concerns in four studies,^30^, ^31^, ^32^, ^33^ and in de Boer et al.^33^, because they used the follow‐up as a reference.

### 3.2 Individual and summary estimates of test accuracy

**FIGURE 2:**
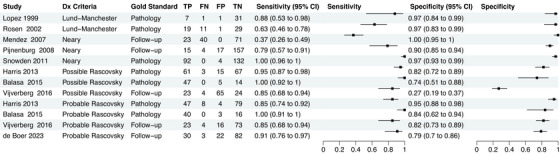
Forest plots showing the individual sensitivity and specificity values for each included study. Forest plot displaying individual sensitivity and specificity estimates for the diagnostic criteria evaluated in the included studies. The diagnostic accuracy of Lund‐Manchester, Neary, and Rascovsky (possible and probable) criteria is presented, with corresponding 95% confidence intervals. CI, confidence interval; FN, false negative; FP, false positive; TN, true negative; TP, true positive.

**FIGURE 3:**
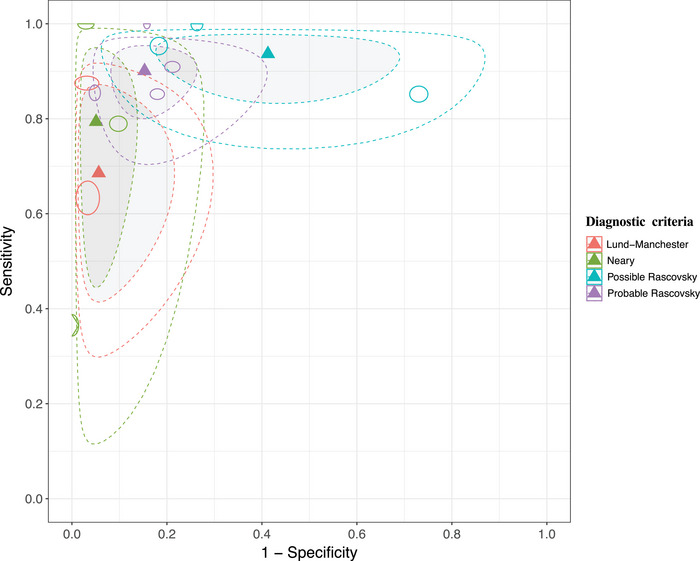
Receiver operating characteristic graphs. The accuracy summary points of Lund‐Manchester (red), Neary (green), possible Rascovsky (blue), and probable Rascovsky criteria (purple) are displayed accordingly. The gray areas surrounded by dotted lines represent the 95% credible region from the bivariate model. The white areas surrounded by dotted lines represent the 95% prediction region from the bivariate model. Circles represent each individual study accordingly to the diagnostic criteria applied, with circle size reflecting study sample size.

Individual sensitivity and specificity values from the included studies are presented in Figure 2. The overall accuracies of bvFTD clinical diagnosis calculated by the random effect bivariate models are presented in Table 2. The chronological analysis of the criteria indicates an improvement in the sensitivity and its precision over time with a summary sensitivity of 0.70 (95% CI: 0.41–0.89), 0.80 (95% CI: 0.48–0.95), 0.94 (95% CI: 0.83–0.98), and 0.90 (95% CI: 0.81–0.95) for Lund‐Manchester,^19^ Neary,^18^ and possible and probable Rascovsky,^6^ respectively. On the other hand, Lund‐Manchester^19^ and Neary^18^ presented higher specificities compared to both Rascovsky criteria.^6^ However, their +LR and −LR had wider confidence intervals, indicating less precision in their estimates (Table 2). Uncertainty can be seen in Figure 3, which displays the summary accuracy points for each criterion and each study and shows larger confidence and prediction regions in Lund‐Manchester,^19^ Neary,^18^ and possible Rascovsky compared to probable Rascovsky^6^ with smaller confidence and prediction regions.

**TABLE 2:** Diagnostic accuracy meta‐analysis bivariate model results of bvFTD clinical diagnosis criteria.

	Number of studies (participants)	Number with bvFTD (%)	Summary sensitivity (95% CI)	Summary specificity (95% CI)	LR + (95% CI)	LR − (95% CI)
Lund‐Manchester	2(100)	38 (17.27%)	0.70 (0.41–0.89)	0.94 (0.79–0.99)	11.95 (2.75–49.76)	0.33 (0.12–0.65)
Neary	3 (555)	174 (31.35%)	0.80 (0.48–0.95)	0.95 (0.85–0.98)	15.38 (4.57–43.37)	0.21 (0.06–0.57)
Possible Rascovsky	3 (328)	138 (42.07%)	0.94 (0.83–0.98)	0.60 (0.31–0.82)	2.31 (1.32–5.09)	0.11 (0.04–0.37)
Probable Rascovsky	4 (450)	155 (34.44%)	0.90 (0.81–0.95)	0.85 (0.73–0.92)	5.88 (3.21–10.78)	0.12 (0.06–0.23)

*Notes*: The addition of number of participants and participants with bvFTD in this table do not represent the overall number of participants in these studies or with bvFTD diagnosis due to overlap of the sample in studies assessing both possible and probable Rascovsky criteria.

Abbreviations: bvFTD, Behavioral variant frontotemporal dementia; CI, confidence intervals; LR, likelihood ratio.

### 3.3 Assessment of heterogeneity

Visual inspection of individual studies suggests the presence of heterogeneity indexed by asymmetry among the 95% CI of individual studies (Figure 2). A priori possible sources of heterogeneity were the study setting, study design, reference test (pathology vs. follow‐up), mean age of participants, and age of onset. We did not perform subgroup analysis by population setting because all studies were hospital based and by participants’ characteristics and for study design, age of participants, and age of onset because of insufficient data. Visual inspection of forest plots by reference standard are displayed in Figure S2 in supporting information. Due to low number of included articles, we were able to create a subgroup analysis comparing follow‐up and pathology only for probable Rascovsky^6^ using the “imperfect gold standard” function of MetaBayesDTA (Figure S3 in supporting information). This function allows us to compare and calculate the sensitivity and specificity estimates for the reference tests.^25^ Studies using pathology as a reference standard presented a higher sensitivity than follow‐up (0.90, 95% CI: 0.70–0.97 vs. 0.61, 95% CI: 0.28–0.91, respectively) while follow‐up studies had a larger specificity (pathology: 0.88, 95% CI: 0.68–0.97 vs. follow‐up 0.93, 95% CI: 0.77–0.98). However, studies using pathology suggest better accuracy than follow‐up.

### 3.4 Sensitivity analysis and publication bias

Leave‐one‐out analysis did not show any study significantly influencing the pooled estimates (Figures S4–S6 in supporting information). Other intended sensitivity analyses and publication bias assessments were not conducted due to the small number of studies.

## 4 DISCUSSION

In this systematic review and meta‐analysis, we evaluated the diagnostic accuracy of clinical criteria for bvFTD across nine studies involving 1130 patients. Our findings indicate that refinement of bvFTD criteria has increased the sensitivity of bvFTD diagnosis, particularly with the introduction of the Rascovsky criteria.^6^ The summary sensitivity increased from 0.70 (95% CI, 0.41–0.89) for the Lund‐Manchester criteria^19^ to 0.90 (95% CI, 0.81–0.95) for the probable bvFTD Rascovsky criteria.^6^ However, specificity decreased with the more recent Rascovsky criteria,^6^ despite its improved −LR and more precise +LR.

The observed improvement in sensitivity likely reflects the evolution and refinement of diagnostic criteria for bvFTD, potentially driven by significant advances in the understanding of the disease's clinical and pathological features. Early criteria, such as the Lund‐Manchester^19^ and Neary criteria,^18^ were stringent and required the presence of multiple core features for diagnosis, which may have limited their sensitivity. The Rascovsky criteria,^6^ introduced in 2011, offered a more flexible approach by allowing a diagnosis of possible bvFTD when any three of six behavioral or cognitive symptoms are present and probable bvFTD when these symptoms are accompanied by functional decline and consistent neuroimaging findings. This flexibility likely contributes to the higher sensitivity observed with the Rascovsky criteria.

Despite the increased sensitivity, the decreased specificity with the newer criteria highlights the ongoing challenges in accurately diagnosing bvFTD. The overlap of bvFTD symptoms with those of psychiatric disorders and other neurodegenerative diseases, such as AD, contributes to diagnostic complexity. In our analysis, psychiatric disorders and AD were the most common sources of misdiagnosis. This finding aligns with previous studies indicating that ≈ 50% of bvFTD patients initially receive a psychiatric diagnosis before being correctly identified with bvFTD.^37^ The decreased specificity may reflect the broader inclusion criteria of the Rascovsky guidelines, which, while capturing bvFTD cases, also encompass individuals with overlapping symptoms from other conditions. The increased precision of the positive and negative likelihood ratios with the Rascovsky criteria suggests that, despite lower specificity, these criteria provide a more reliable diagnostic framework. Furthermore, the results of our meta‐analysis are consistent with the findings of the seminal study by Rascovsky et al.,^6^ which showed that the sensitivity of the FTDC criteria was markedly higher than that of the Neary criteria. Addressing bvFTD phenocopies, which mimic the clinical presentation of bvFTD without underlying neurodegeneration, is critical for improving diagnostic accuracy. The integration of reliable biomarkers reflecting underlying pathophysiological mechanisms—such as advanced neuroimaging techniques or fluid‐based biomarkers—could provide essential tools to enhance specificity, reduce diagnostic uncertainty, and better distinguish true bvFTD cases from phenocopies. In addition, recent analyses have highlighted that the cognitive criterion of the Rascovsky criteria may be disproportionately restrictive, particularly when deficits extend to episodic memory or visuospatial domains.^38^ This limitation further underscores the need for refinement of the clinical criteria to balance sensitivity with specificity and improve diagnostic accuracy in real‐world practice.

As expected, our analysis revealed that diagnostic accuracy was dependent on the reference standard. In particular, we found a higher sensitivity in pathology with respect to clinical follow‐up. Pathological confirmation remains the gold standard for diagnosing bvFTD, providing definitive evidence of FTLD.^6^ However, access to neuropathological examination is often limited to specialized centers, typically university‐affiliated hospitals with research capabilities. On the other hand, while follow‐up as reference standard provides an alternative for settings without access to pathological evaluation, diagnosis ascertainment may vary within and between studies, as it is susceptible to the context and expertise of the assessor, which could increase the risk of misclassification.^39^ Despite all included studies being hospital‐based and primarily conducted in tertiary care or university centers, the clinical criteria evaluated are fundamental procedures to achieve tally based on clinical features and supported by neuroimaging studies that are widely available. Therefore, these criteria are applicable across various health‐care settings, including community and primary care environments, enhancing their utility in routine clinical practice. The mean age of onset reported across studies was ≈ 60 years, consistent with epidemiological data on bvFTD incidence, reinforcing the relevance of our findings.

This study has some limitations. The small number of included studies and relatively limited sample sizes reflect the rarity of bvFTD and the challenges associated with obtaining pathological confirmation. The limited number of studies also reflects the fact that only a minority fulfilled the methodological requirements for diagnostic test accuracy meta‐analysis as outlined in the Cochrane Handbook.^21^ The heterogeneity among studies, evident from variations in study design, patient populations, and reference standards, may affect the generalizability of our findings. Another source of heterogeneity is the evolution of neuropathological assessment over time, with progressively refined criteria for FTLD.^3^, ^17^, ^40^ Differences in case mix also contributed, with some cohorts enriched for late‐onset psychiatric presentations and others for neurodegenerative cases, which may have differentially influenced sensitivity and specificity. Furthermore, the diagnostic criteria were developed and validated in different time periods, and the comparison across them may also reflect broader temporal changes in the field rather than intrinsic differences alone. Additionally, there was a lack of diversity in study populations. Although no study reported information regarding race and ethnicity, considering the geographical location of the studies it is probable that most participants were White, with Asian and Black individuals significantly underrepresented.^41^ This demographic limitation restricts the applicability of our conclusions across different racial and ethnic groups. As mentioned, the reliance on studies conducted in specialized centers may limit the applicability of findings to broader clinical settings.

Despite these limitations, our study represents the first attempt to meta‐analyze the accuracy of clinical diagnostic criteria for bvFTD. Although biomarkers are in continuous development, it is unlikely that a biological diagnosis will be feasible for all patients in the near future, partially because bvFTD encompasses a spectrum of underlying pathologies, including TDP‐43 and tau proteinopathies.^16^ Furthermore, while the genetics of bvFTD are expanding—with more mutations accounting for previously unexplained heritability—clinical criteria remain the cornerstone for diagnosis. Therefore, our findings underscore the importance of clinical diagnostic criteria and support their continued use as the gold standard for diagnosing bvFTD in clinical practice.

Our systematic review and meta‐analysis demonstrates that the sensitivity of bvFTD clinical diagnostic criteria has improved with a better understanding of the disease and refinement of diagnostic criteria, in particular with the adoption of the Rascovsky criteria. However, this improvement in sensitivity comes with a decrease in specificity, highlighting the inherent challenges in differentiating bvFTD from other neurodegenerative and psychiatric disorders. These findings underscore the need for better diagnostic tools such as biomarkers reflecting pathogenic mechanisms and ongoing pathophysiological processes. Until such advancements are realized, clinical criteria along with neuroimaging techniques, remain essential for the diagnosis of bvFTD across various health‐care settings.

## CONFLICT OF INTEREST STATEMENT

Nothing to report. Author disclosures are available in the Supporting Information.

## CONSENT STATEMENT

Ethical approval and patient consent were not required as this study is a systematic review of previously published literature and does not involve any original data collection from human participants.

## Supporting information



Supporting information

Supporting information
