# Supplementary material for: Accuracy of clinical diagnosis of behavioral variant frontotemporal dementia: A systematic review and meta‐analysis
Source: Alzheimers Dement (Amst). 2026 Feb 28;18(1):e70266. doi: 10.1002/dad2.70266 (PMC12949661; doi:10.1002/dad2.70266)
Supplement: Supplementary file 1 — Supporting information [file DAD2-18-e70266-s001.docx]

**SUPPLEMENT**

**eTable 1.** PRISMA checklist

| **Section/Topic** | **#** | **Checklist item** | **Page #** |
| --- | --- | --- | --- |
| **TITLE** |  |  |  |
| Title | 1 | Identify the report as a systematic review, meta-analysis, or both. | 1 |
| **ABSTRACT** |  |  |  |
| Structured summary | 2 | Provide a structured summary including, as applicable: background; objectives; data sources; study eligibility criteria, participants, and interventions; study appraisal and synthesis methods; results; limitations; conclusions and implications of key findings; systematic review registration number. | 3 |
| **INTRODUCTION** |  |  |  |
| Rationale | 3 | Describe the rationale for the review in the context of what is already known. | 5 |
| Objectives | 4 | Provide an explicit statement of questions being addressed with reference to participants, interventions, comparisons, outcomes, and study design (PICOS). | 6 |
| **METHODS** |  |  |  |
| Protocol and registration | 5 | Indicate if a review protocol exists, if and where it can be accessed (e.g., Web address), and, if available, provide registration information including registration number. | 6 |
| Eligibility criteria | 6 | Specify study characteristics (e.g., PICOS, length of follow- up) and report characteristics (e.g., years considered, language, publication status) used as criteria for eligibility, giving rationale. | 7 |
| Information sources | 7 | Describe all information sources (e.g., databases with dates of coverage, contact with study authors to identify additional studies) in the search and date last searched. | 7-8 |
| Search | 8 | Present full electronic search strategy for at least one database, including any limits used, such that it could be repeated. | Suppl. table 2 |
| Study Selection | 9 | State the process for selecting studies (i.e., screening, eligibility, included in systematic review, and, if applicable, included in the meta-analysis). | 8 |
| **Section/Topic** | **#** | **Checklist item** | **Page #** |
| Data collection process | 10 | Describe method of data extraction from reports (e.g., piloted forms, independently, in duplicate) and any processes for obtaining and confirming data from investigators. | 8 |
| Data items | 11 | List and define all variables for which data were sought (e.g., PICOS, funding sources) and any assumptions and simplifications made. | 8 |
| Risk of bias in individual studies | 12 | Describe methods used for assessing risk of bias of individual studies (including specification of whether this was done at the study or outcome level), and how this information is to be used in any data synthesis. | 8-9 |
| Summary measures | 13 | State the principal summary measures (e.g., risk ratio, difference in means). | 8-9 |
| Synthesis of results | 14 | Describe the methods of handling data and combining results of studies, if done, including measures of consistency (e.g., I^2^) for each meta-analysis. | 9 |
| Risk of bias across studies | 15 | Specify any assessment of risk of bias that may affect the cumulative evidence (e.g., publication bias, selective reporting within studies). | 8-9 |
| Additional analyses | 16 | Describe methods of additional analyses (e.g., sensitivity or subgroup analyses, meta-regression), if done, indicating which were pre-specified. | 9-10 |
| **RESULTS** |  |  |  |
| Study selection | 17 | Give numbers of studies screened, assessed for eligibility, and included in the review, with reasons for exclusions at each stage, ideally with a flow diagram. | 10 |
| Study characteristics | 18 | For each study, present characteristics for which data were extracted (e.g., study size, PICOS, follow-up period) and provide the citations. | 10-11 |
| Risk of bias within studies | 19 | Present data on risk of bias of each study and, if available, any outcome level assessment (see item 12). | 11-12 |
| Results of individual studies | 20 | For all outcomes considered (benefits or harms), present, for each study: (a) simple summary data for each intervention group (b) effect estimates and confidence intervals, ideally with a forest plot. | 12 and table 1 |
| **Section/Topic** | **#** | **Checklist item** | **Page #** |
| Synthesis of results | 21 | Present results of each meta-analysis done, including confidence intervals and measures of consistency. | 12, figures 2 and 3 |
| Risk of bias across studies | 22 | Present results of any assessment of risk of bias across studies (see Item 15). | 12-13 |
| Additional analysis | 23 | Give results of additional analyses, if done (e.g., sensitivity or subgroup analyses, meta-regression [see Item 16]). | 12-13 |
| **DISCUSSION** |  |  |  |
| Summary of evidence | 24 | Summarize the main findings including the strength of evidence for each main outcome; consider their relevance to key groups (e.g., healthcare providers, users, and policy makers). | 14-16 |
| Limitations | 25 | Discuss limitations at study and outcome level (e.g., risk of bias), and at review-level (e.g., incomplete retrieval of identified research, reporting bias). | 16 |
| Conclusions | 26 | Provide a general interpretation of the results in the context of other evidence, and implications for future research. | 16-17 |
| Funding | 27 | Describe sources of funding for the systematic review and other support (e.g., supply of data); role of funders for the systematic review. | 17 |

**eTable 2.** Search strategies

| **Database** | **Search Terms** | **Results** |
| --- | --- | --- |
| **MEDLINE**  **Pubmed**  **(01/05/24)** | ("frontotemporal dementia behavioral variant"[Title/Abstract] OR (("frontotemporal dementia"[MeSH Terms] OR ("Frontotemporal"[All Fields] AND "dementia"[All Fields]) OR "frontotemporal dementia"[All Fields]) AND "behavioural variant"[Title/Abstract]) OR "Neary"[Text Word] OR "Rascovsky"[Text Word] OR "frontotemporal lobar degeneration"[MeSH Terms])  AND  ("diagnosis"[MeSH Terms] OR "diagnostic accuracy"[Title/Abstract] OR "sensitivity"[Title/Abstract] OR "specificity"[Title/Abstract] OR "positive predictive value"[Title/Abstract] OR "negative predictive value"[Title/Abstract] OR "accuracy"[Title/Abstract] OR "predictive value"[Title/Abstract] OR "accurate"[Title/Abstract] OR "diagnostic criteria"[Title/Abstract] OR "false positive"[Title/Abstract] OR "false negative"[Title/Abstract]) | 2556 |
| **Web of Science**  **(01/05/24)** | (TS=(frontotemporal dementia) OR TS=(frontotemporal lobar degeneration) OR AU=(Neary D) OR AU=(Rascovsky K) OR TS=(frontotemporal dementia behavioral variant))  AND  (TS=(diagnostic accuracy) OR TS=(Sensitivity) OR TS=(Specificity) OR TS=(Positive predictive value) OR TS=(Negative predictive value) OR TS=(Accuracy) OR TS=(Predictive value) OR TS=(False Positive) OR TS=(False negative) OR TS=(Diagnostic criteria)) | 3168 |
| **Embase**  **(01/05/24)** | 'frontotemporal dementia':ti,ab,kw OR rascovsky:au OR neary:au OR 'frontotemporal dementia behavioral variant':ti,ab,kw OR 'frontotemporal lobe degeneration':ti,ab,kw  AND  'diagnostic accuracy':ti,ab,kw OR sensitivity:ti,ab,kw OR specificity:ti,ab,kw OR 'positive predictive value':ti,ab,kw OR 'negative predictive value':ti,ab,kw OR accuracy:ti,ab,kw OR 'predictive value':ti,ab,kw OR 'false positive':ti,ab,kw OR 'false negative':ti,ab,kw OR 'diagnostic criteria':ti,ab,kw | 1855 |
| **Database** | **Search Terms** | **Results** |
| **LILACS**  **(01/05/24)** | (“frontotemporal dementia” OR rascovsky OR neary OR “frontotemporal dementia behavioral variant” OR “frontotemporal lobe degeneration”)      AND (("diagnostic accuracy" OR "Sensitivity" OR “Specificity” OR "Positive Predictive Value" OR "Negative Predictive Value"OR "Accuracy" OR "Predictive Value" OR "predictive values" OR "diagnostic criteria" OR "Criteria" OR "false positive" OR "false negative")) | 835 |

**eTable 3.** Articles Excluded by full-text screening and reasons for exclusion.

| **N°** | **Author** | **Title** | **Journal** | **Reason of exclusion** |
| --- | --- | --- | --- | --- |
| 1 | Adams 2016 | Do criteria for behavioural variant frontotemporal dementia capture the cognitive and behavioural profile of ALS/FTD? | Journal of Neurochemistry | Conference Abstract |
| 2 | Andersson 2020 | Clinicopathological concordance in cognitive disease diagnostics | Clinical Neuropathology | Wrong study design |
| 3 | Barker 2022 | Proposed research criteria for prodromal behavioural variant frontotemporal dementia. | Brain | Wrong patient population |
| 4 | Borroni 2015 | Early stage of behavioral variant frontotemporal dementia: clinical and neuroimaging correlates. | Neurobiol Aging | No Gold Standard |
| 5 | CalifNon-ADReliabilConsortium 2007 | Inter-rater reliability of diagnostic criteria for frontotemporal dementia (FTD): Findings from the California Non-AD diagnostic reliability consortium | NEUROLOGY | Wrong study design |
| 6 | Chare 2014 | New criteria for frontotemporal dementia syndromes: Clinical and pathological diagnostic implications | Journal of Neurology, Neurosurgery and Psychiatry | Wrong study design |

| **N°** | **Author** | **Title** | **Journal** | **Reason of exclusion** |
| --- | --- | --- | --- | --- |
| 7 | Costa 2012 | Diagnostic criteria for the behavioural variant of frontotemporal dementia-revision of two criteria system | Journal of Neurology | Conference Abstract |
| 8 | Costa 2013 | Comparison of 2 diagnostic criteria for the behavioral variant of frontotemporal dementia | American Journal of Alzheimer's Disease and other Dementias | Only sensitivity |
| 9 | DeSanchez 2011 | Frontotemporal lobar degeneration: Diagnosis and epidemiology in a colombian memory clinic through thirteen years (1997-2010) | Alzheimer's and Dementia | Conference Abstract |
| 10 | Devenney 2015 | Progression in Behavioral Variant Frontotemporal Dementia: A Longitudinal Study. | JAMA Neurol | Only sensitivity |
| 11 | Gall 2012 | Sensitivity and specificity of behavioural variant frontotemporal dementia criteria | Dementia and Geriatric Cognitive Disorders | Conference Abstract |

| **N°** | **Author** | **Title** | **Journal** | **Reason of exclusion** |
| --- | --- | --- | --- | --- |
| 12 | Ghosh 2013 | Using the revised diagnostic criteria for frontotemporal dementia in India: evidence of an advanced and florid disease. | PLoS One | Wrong study design |
| 13 | Giannini 2016 | Clinicopathological correlations of ad neuropathology in the logopenic variant of primary progressive aphasia | Neurology | Wrong patient population |
| 14 | Gislason 2015 | Effect of diagnostic criteria on prevalence of frontotemporal dementia in the elderly. | Alzheimers Dement | Only sensitivity |
| 15 | Grossman 2007 | Distinct antemortem profiles in patients with pathologically defined frontotemporal dementia | Archives of Neurology | Diagnostic criteria not defined |
| 16 | Grossman 2008 | Longitudinal decline in autopsy-defined frontotemporal lobar degeneration | NEUROLOGY | Wrong study design |
| 17 | Guimaraes 2013 | Analysis of a case series of behavioral variant frontotemporal dementia: emphasis on diagnostic delay | Dement. neuropsychol | Only sensitivity |

| **N°** | **Author** | **Title** | **Journal** | **Reason of exclusion** |
| --- | --- | --- | --- | --- |
| 18 | Halliday 2002 | Consensus neuropathological diagnosis of common dementia syndromes: Testing and standardising the use of multiple diagnostic criteria | Acta Neuropathologica | Wrong study design |
| 19 | Harris 2012 | Specificity of pathological diagnoses in subtypes of primary progressive aphasia (PPA): An evaluation of the 2011 PPA criteria | Dementia and Geriatric Cognitive Disorders | Conference Abstract |
| 20 | Harris 2012 | Comparing the sensitivity and specificity of old and new criteria for frontotemporal dementia | Dementia and Geriatric Cognitive Disorders | Conference Abstract |
| 21 | Harris 2013 | Classification and pathology of primary progressive aphasia | NEUROLOGY | Wrong patient population |
| 22 | Harris 2015 | Do NIA-AA criteria distinguish Alzheimer's disease from frontotemporal dementia? | Alzheimer's and Dementia | Wrong study design |
| 23 | Kaneta 2014 | Comparison of diagnostic accuracy of AD at patients' first contact between visual assessment and automated analysis of brain perfusion SPECT | Alzheimer's and Dementia | Conference Abstract |
| **N°** | **Author** | **Title** | **Journal** | **Reason of exclusion** |
| 24 | Kertesz 2008 | Validation of diagnostic criteria for frontotemporal dementia | NATURE CLINICAL PRACTICE NEUROLOGY | Letter to the editor/ comment |
| 25 | Knopman 2005 | Antemortem diagnosis of frontotemporal lobar degeneration | Annals of Neurology | Diagnostic criteria not defined |
| 26 | Krudop 2014 | Building a new paradigm for the early recognition of behavioral variant frontotemporal dementia: Late Onset Frontal Lobe Syndrome study. | Am J Geriatr Psychiatry | Wrong study design |
| 27 | Krudop 2015 | Identifying bvFTD Within the Wide Spectrum of Late Onset Frontal Lobe Syndrome: A Clinical Approach. | Am J Geriatr Psychiatry | Wrong study design |
| 28 | Krudop 2016 | The pitfall of behavioral variant frontotemporal dementia mimics despite multidisciplinary application of the FTDC criteria | Journal of Neurochemistry | Conference Abstract |
| 29 | Lamarre 2013 | Interrater reliability of the new criteria for behavioral variant frontotemporal dementia. | Neurology | Wrong study design |

| **N°** | **Author** | **Title** | **Journal** | **Reason of exclusion** |
| --- | --- | --- | --- | --- |
| 30 | Mariani 2006 | Frontotemporal dementia | Neurological Sciences | Wrong study design |
| 31 | Mendez 2004 | The accuracy of clinical criteria for the diagnosis of frontotemporal dementia | International Journal of Psychiatry in Medicine | Letter to the editor/comment |
| 32 | Mendez 2010 | Prevalence of new and predominant presenting criteria among patients with behavioral variant frontotemporal dementia (bvFTD) | Dementia and Geriatric Cognitive Disorders | Conference Abstract |
| 33 | Mendez 2013 | Clinicopathologic differences among patients with behavioral variant frontotemporal dementia. | Neurology | Wrong study design |
| 34 | Musa 2020 | Alzheimer's Disease or Behavioral Variant Frontotemporal Dementia? Review of Key Points Toward an Accurate Clinical and Neuropsychological Diagnosis. | J Alzheimers Dis | Wrong study design |
| 35 | Neary 1998 | Frontotemporal lobar degeneration: A consensus on clinical diagnostic criteria | Neurology | Wrong study design |
| 36 | Perry 2014 | Clinicopathological correlations in behavioral variant frontotemporal dementia | American Journal of Neurodegenerative Diseases | Only sensitivity |

| **N°** | **Author** | **Title** | **Journal** | **Reason of exclusion** |
| --- | --- | --- | --- | --- |
| 37 | Perry 2019 | Factors that predict diagnostic stability in neurodegenerative dementia. | J Neurol | Wrong study design |
| 38 | Piguet 2009 | Sensitivity of current criteria for the diagnosis of behavioral variant frontotemporal dementia | Neurology | Only sensitivity |
| 39 | Piguet 2011 | Clinical phenotypes in autopsy-confirmed pick disease | Neurology | Only sensitivity |
| 40 | Pijnenburg 2018 | IDENTIFYING BEHAVIORAL VARIANT FRONTOTEMPORAL DEMENTIA AMONG PATIENTS WITH A LATE-ONSET FRONTAL LOBE SYNDROME: SUMMARY RESULTS OF THE LOF STUDY | Alzheimer's and Dementia | Diagnostic criteria not defined |
| 41 | Qian 2016 | Misdiagnosis of Alzheimer's disease: Inconsistencies between clinical diagnosis and neuropathological confirmation | Alzheimer's and Dementia | Conference Abstract |
| 42 | Rascovsky 2010 | Sensitivity of consensus diagnostic criteria in autopsy-confirmed patients with behavioral variant frontotemporal dementia (bvFTD): First report of the international bvFTD criteria Consortium | Dementia and Geriatric Cognitive Disorders | Conference Abstract |

| **N°** | **Author** | **Title** | **Journal** | **Reason of exclusion** |
| --- | --- | --- | --- | --- |
| 43 | Rascovsky 2011 | Sensitivity of revised diagnostic criteria for the behavioural variant of frontotemporal dementia. | Brain | Only sensitivity |
| 44 | Scarioni 2018 | Diagnosing frontotemporal dementia: Clinico-pathological correlations in the Dutch population | European Journal of Neurology | Diagnostic criteria not defined |
| 45 | Selvackadunco 2018 | Comparison of clinical and neuropathological diagnoses of neurodegenerative diseases in two centres from the Brains for Dementia Research (BDR) cohort | Neuropathology and Applied Neurobiology | Diagnostic criteria not defined |
| 46 | Shinagawa 2016 | When a Little Knowledge Can Be Dangerous: False-Positive Diagnosis of Behavioral Variant Frontotemporal Dementia among Community Clinicians. | Dement Geriatr Cogn Disord | Diagnostic criteria not defined |
| 47 | Shinagawa 2022 | The Diagnostic Patterns of Referring Physicians and Hospital Expert Psychiatrists Regarding Particular Frontotemporal Lobar Degeneration Clinical and Neuropathological Subtypes | Journal of Alzheimer's Disease | Wrong study design |

| **N°** | **Author** | **Title** | **Journal** | **Reason of exclusion** |
| --- | --- | --- | --- | --- |
| 48 | Solje 2015 | The Phenotype of the C9ORF72 Expansion Carriers According to Revised Criteria for bvFTD. | PLoS One | Only sensitivity |
| 49 | Straub 2016 | Multivariate diagnostic approaches in Frontotemporal lobar degeneration-Data from the german FTLDc | Journal of Neurochemistry | Diagnostic criteria not defined |
| 50 | Sutovsky 2014 | Clinical accuracy of the distinction between Alzheimer's disease and frontotemporal lobar degeneration | Bratislava Medical Journal | Wrong study design |
| 51 | Vandenberghe 2011 | Sense and sensitivity of novel criteria for frontotemporal dementia | Brain | Wrong study design |
| 52 | Varma 1999 | Evaluation of the NINCDS-ADRDA criteria in the differentiation of Alzheimer's disease and frontotemporal dementia | Journal of Neurology Neurosurgery and Psychiatry | Wrong study design |
| 53 | Vijverberg 2015 | Frontotemporal dementia and related disorders: Diagnostic accuracy of the FTD consensus criteria in the late onset frontal lobe syndrome | International Psychogeriatrics | Conference Abstract |

| **N°** | **Author** | **Title** | **Journal** | **Reason of exclusion** |
| --- | --- | --- | --- | --- |
| 54 | Vijverberg 2017 | The Diagnostic Challenge of the Late-Onset Frontal Lobe Syndrome: Clinical Predictors for Primary Psychiatric Disorders Versus Behavioral Variant Frontotemporal Dementia. | J Clin Psychiatry | Duplicate population |
| 55 | Zhutovsky 2019 | Individual Prediction of Behavioral Variant Frontotemporal Dementia Development Using Multivariate Pattern Analysis of Magnetic Resonance Imaging Data. | J Alzheimers Dis | Wrong study design |

**eTable 4.** Tailored Quality Assessment of Diagnostic Accuracy (QUADAS-2) tool.

| **Risk factor** | **Domain** | **Questions** | **Observations** |
| --- | --- | --- | --- |
|  | **Patient selection** | Was a consecutive or random sample of patients enrolled? |  |
|  |  | Was a case-control design avoided? |  |
|  |  | Did the study avoid inappropriate exclusions? |  |
|  | **Index Test** | Were the index test results interpreted without knowledge of the results of the reference standard? (Blinding) |  |
|  |  | If a threshold was used, was it prespecified? | Question removed |
|  | **Reference Standard** | Is the reference standard likely to correctly classify the target condition? |  |
|  |  | Were the reference standard results interpreted without knowledge of the results of the index test? |  |
|  | **Flow and Timing** | Was the follow-up of at least 2 years? | ONLY for those with follow-up as gold standard. |
|  |  | Did all patients receive the same reference standard? |  |
|  |  | Were all patients included in the analysis? |  |

| **Risk factor** | **Domain** | **Questions** | **Observations** |
| --- | --- | --- | --- |
| **Applicability** | **Patient Selection** | Are there concerns that the included patients and setting do not match with systematic review question? |  |
|  |  | Are there concerns that the included patients and setting do not match with systematic review question? |  |
| **Applicability** | **Domain** | **Questions** | **Observations** |
|  | **Index Test** | Are there concerns that the index test, its conduct, or its interpretation differs from the review question? |  |
|  | **Reference Standard** | Are there concerns that the target condition as defined by the reference standard does not match the question? |  |

**eTable 5.** False-negative and false-positive diagnosis of bvFTD

| **Study ID** | **Dx. Criteria** | **Missdiagnosis** |
| --- | --- | --- |
| Lopez 1999 | Lund-Manchester | **False-negative cases:**  -Creutzfeld-Jakob disease (n=1)  -Probable AD(n=3)  -Possible AD (n=1)  -Normal Pressure hydrocephalus (n=1)  **False-positive cases:**  -Not reported |
| Rosen 2002 | Lund-Manchester | **False-negative cases:**  Control (n=1)  AD (n=7)  AD + CVD (n=2)  Other=1  **False-positive cases:**  AD + CVD=1  AD + DLB=1 |
| Mendez 2007 | Neary | **False-negative cases:**  Not reported  **False-positive cases:**  -Depression/bipolar disorder (n=15)  -Atypical psychosis (n=7)  -Anxiety disorder (n=5)  -Personality disorder (n=5)  -Adjustment reaction (n=4)  -AD (n=17)  -Other neurologic disorders (n=9)  -Anoxic encephalopathy (n=2)  -Creutzfeldt-Jakob disease (n=2)  -Hashimoto encephalopathy (n=1)  -Neurosarcoidosis (n=1)  -Normal pressure hydrocephalus (n=1)  -Paraneoplastic syndrome (n=1)  -Sleep apnea syndrome (n=1)  -Uncertain or undiagnosed (n=9) |
| Pijnenburg 2008 | Neary | Not reported |
| Snowden 2011 | Neary | **False-negative cases:**  -AD (n=2)  -CVD (n=1)  -Other (n=1)  **False-postive cases:**  None |

| **Study ID** | **Dx. Criteria** | **Missdiagnosis** |
| --- | --- | --- |
| Harris 2013 | Rascovsky | **False-negative cases:**  -ALS (n=1)  -Limbic/temporal presentation of AD (n=1)  -Clinical diagnosis of FTD (did not fulfil criteria)  **False-positive cases:**  -AD (n=9)  -Mixed AD and DLB (n=1)  -Prion disease (n=1)  -CVD (n=2)  -Nonspecific changes (n=1) |
| Balasa 2015 | Rascovsky | **False-negative cases:**  -AD (n=3)  - Acute psychotic episode possibly due to seronegative encephalitis (n=1)  **False-positive cases:**  -Advanced AD changes with neocortical Lewy Bodies.  -Orthochromatic pigmentary leukodystrophy with no FTLD or AD lesions.  -Diffuse small vessel disease associated with a Braak stage III of neurofibrillary pathlogy and a moderate density of neuritic plaques.  -Extensive beta-amyloid and neurofibrillary pathology compatible with AD dx and scarse TDP43 protein inclusions in hippocamus and entorhinal cortex.  -Severe AD pathology with freq. neuritic plaques and extensive neurofibrillary tangles with intense amyloid angiopathy and small vessel disease. |
| Vijverberg 2016 | Rascovsky | **False-negative cases:**  Not reported  **False-positive cases:**  -Psychiatric disorders (n=15)  -Other neurodegenerative disorders (n=7)  -Other neurological disorders (n=3) |

| **Study ID** | **Dx. Criteria** | **Missdiagnosis** |
| --- | --- | --- |
| De Boer 2023 | Rascovsky | **False-negative cases:**  -Primary psychiatric disorder (n=1).  **False-positive cases:**  -Primary psychiatric disorder (n=12)  - Other neurological disorder (n=6) |
| AD: Alzheimer’s disease; ALS: Amyotrophic Lateral Sclerosis; CVD: Cerebrovascular dementia; DLB: Dementia Lewy Bodies; FTD: Frontotemporal dementia; FTLD: Fronto-temporal Lobar Degeneration; TDP43: Transactive response DNA binding protein of 43 kDa. | | |

**eFIGURES**

**eFigure 1.** Risk of bias and applicability Assessment performed with a tailored version of the QUADAS-2 tool.

**eFigure 2.** Forest plot showing the subgroup analysis by reference standard.

**eFigure 3.** Receiver operative characteristic graphs showing the accuracy summary points of Follow-up, pathology, and overall index criteria for probable Rascovsky only. The grey areas surrounded by dotted lines represent the 95% credible region from the bivariate model. The white areas surrounded by dotted lines represent the 95% prediction region from the bivariate model.In some cases they may overalap. Small red dots correspond to each single article using probable Rascovksy criteria.

**eFigure 4.** Forest plot showing the sensitivity analysis of Neary criteria assessing individual study effects comparing the summary sensitivity and specificity by excluding one study at the time.

**eFigure 5.** Forest plot showing the sensitivity analysis of possible Rascovsky criteria assessing individual study effects comparing the summary sensitivity and specificity by excluding one study at the time.

**eFigure 6.** Forest plot showing the sensitivity analysis of probable Rascovsky criteria assessing individual study effects comparing the summary sensitivity and specificity by excluding one study at the time.
